# The importance of aboveground–belowground interactions on the evolution and maintenance of variation in plant defense traits

**DOI:** 10.3389/fpls.2013.00431

**Published:** 2013-11-28

**Authors:** Moniek van Geem, Rieta Gols, Nicole M. van Dam, Wim H. van der Putten, Taiadjana Fortuna, Jeffrey A. Harvey

**Affiliations:** ^1^Department of Terrestrial Ecology, Netherlands Institute of Ecology (NIOO-KNAW)Wageningen, Netherlands; ^2^Laboratory of Entomology, Wageningen UniversityWageningen, Netherlands; ^3^Radboud University Nijmegen, Institute for Water and Wetland ResearchNijmegen, Netherlands; ^4^Laboratory of Nematology, Wageningen UniversityWageningen, Netherlands; ^5^Department of Ecological Sciences, Animal Ecology, VU UniversityAmsterdam, Netherlands

**Keywords:** genetic variation, aboveground–belowground interactions, herbivore, natural enemy, plant defense, selection pressures, *Brassica oleracea*

## Abstract

Over the past two decades a growing body of empirical research has shown that many ecological processes are mediated by a complex array of indirect interactions occurring between rhizosphere-inhabiting organisms and those found on aboveground plant parts. Aboveground–belowground studies have thus far focused on elucidating processes and underlying mechanisms that mediate the behavior and performance of invertebrates in opposite ecosystem compartments. Less is known about genetic variation in plant traits such as defense as that may be driven by above- and belowground trophic interactions. For instance, although our understanding of genetic variation in aboveground plant traits and its effects on community-level interactions is well developed, little is known about the importance of aboveground–belowground interactions in driving this variation. Plant traits may have evolved in response to selection pressures from above- and below-ground interactions from antagonists and mutualists. Here, we discuss gaps in our understanding of genetic variation in plant-related traits as they relate to aboveground and belowground multitrophic interactions. When metabolic resources are limiting, multiple attacks by antagonists in both domains may lead to trade-offs. In nature, these trade-offs may critically depend upon their effects on plant fitness. Natural enemies of herbivores may also influence selection for different traits via top–down control. At larger scales these interactions may generate evolutionary “hotspots” where the expression of various plant traits is the result of strong reciprocal selection via direct and indirect interactions. The role of abiotic factors in driving genetic variation in plant traits is also discussed.

## INTRODUCTION

Of the many traits possessed by plants that are closely tied with their growth, survival and fitness, those relating to defence have been especially well studied over many years (see reviews by Karban and Baldwin, 1997; Schoonhoven et al., 2005). Defences in plants are often divided into direct and indirect defenses. Direct defenses are aimed directly at the attackers, such as herbivores, and include morphological (e.g., trichomes or sticky glands) and chemical (toxic secondary compounds) traits that interfere with colonization, feeding, and development of the herbivore. For example, toxic secondary compounds can act as feeding deterrents or negatively alter the performance of a herbivore through increased mortality, slower growth rates, or reduced fitness (Schoonhoven et al., 2005). Indirect defenses are aimed at promoting the efficiency of natural enemies, such as predators or parasitic wasps (parasitoids) that kill the herbivores and thus reduce their damage to the plant. Indirect defenses may also be morphological (e.g., domatia) or chemical (e.g., the production of attractive volatiles, energy sources). Both direct and indirect defenses are expressed constitutively in many plants, meaning that they are always expressed whereas in others they are often inducible, meaning that initial levels are low but increase after attack (Karban and Baldwin, 1997). These traits are often species- (or even genotype) specific, and are assumed to depend on the predictability of attacks from antagonists and susceptibility of plants to these attacks.

Unlike most terrestrial biota, the vast majority of plants occupy two connected “compartments” – the open air and soil – that differ in many biotic and abiotic properties. Aboveground plant structures include stems, branches, leaves, shoots, flowers, and seeds, whereas the soil is dominated by the root system. These differing plant structures facilitate interactions between biotic communities that rarely come into direct physical contact with one another (Soler et al., 2008). In both the soil and aboveground compartments many organisms are associated with the plants, ranging from vertebrates and arthropods to micro-organisms. These organisms may have beneficial, neutral, or negative effects on plant fitness. Plants have evolved a range of strategies to optimize associations with beneficial organisms and/or to prevent or reduce the negative effect of attack from their antagonists. Roots may harbor many antagonists and therefore it is important that plants do not only defend themselves in the shoots but in their roots as well (Bezemer and van Dam, 2005; Van Dam, 2009). In a seminal paper, Ehrlich and Raven (1964) argued that plants and insects are involved in a sequential co-evolutionary arms race in which insect herbivores evolve strategies to deal with plant defences which are countered by new or stronger defences by the plant over evolutionary time. However, at the time their paper was written, the importance of this arms race was restricted to interactions between plants and insects in an aboveground framework. As we will explain here, this “arms race” can also involve interactions between roots and root herbivores as well as indirect interactions involving root and shoot tissues and herbivores feeding on plant structures in opposite compartments.

In this paper we discuss studies investigating the processes and underlying mechanisms that underpin multitrophic interactions with plants in the above- and belowground compartments (hereafter AG and BG). In particular, we broach a topic that has thus far received little attention in the empirical literature: factors generating and maintaining intraspecific variation in plant defense-related traits that are mediated through AG and BG trophic interactions. Plant defense traits often have a genetic basis. The effects of genetic variation in these plant traits on interactions with higher trophic level organisms have primarily been studied for AG plant parts (Crutsinger et al., 2006; Johnson, 2008; Newton et al., 2009a; Utsumi et al., 2011). In the last decade, complexity and ecological realism in experimental designs has increased. This also includes genetic variation in the interacting AG and BG compartments and its effect on interactions with associated organisms (Rasmann et al., 2009; Vandegehuchte et al., 2011). With this paper we make a plea for a more holistic approach with respect to genetic variation and AG–BG interactions. First we give a brief overview of the literature on plant-mediated multitrophic interactions in the AG and BG compartments and the hypotheses and underlying mechanisms that emerged from these studies. We then discuss the role of genetic variation in plant traits in shaping interactions with associated organisms. As an example, we give an overview of current knowledge on inter- and intraspecific AG–BG variation in plant defense traits and their consequences for interactions between insects and plant species in the large family Brassicaceae. We explore how different selection pressures at the species level may lead to the expression of variation in defense traits in roots and shoots using wild cabbage (*Brassica oleracea*) as our model species. We provide new data on root chemical defenses, show how this compares with better-studied AG defenses in this species, and highlight the importance of studying genetic variation in plant traits that play a role in AG and BG interactions with associated organisms in natural systems in order to explain the evolution and maintenance of variation among these traits.

### PLANT MEDIATED ABOVE- AND BELOWGROUND INTERACTIONS: PATTERNS AND HYPOTHESES

Most studies involving plants and their defense traits in a bi- or multitrophic framework have focused on the AG compartment (reviewed by Price et al., 1980; Karban and Baldwin, 1997; Dicke, 1999; Harvey, 2005; Schoonhoven et al., 2005; Ode, 2006; Hopkins et al., 2009). These studies have provided a wealth of data showing that direct and indirect plant defenses can profoundly influence mechanisms governing species-level interactions and the structure of food webs up to (and perhaps even beyond) the fourth trophic level (Harvey et al., 2004, 2007; Bukovinszky et al., 2008). However, it is important to stress that plant-related traits, including defense, can also strongly influence biotic interactions BG (see reviews by Van der Putten et al., 2001; Van Dam et al., 2003; Bezemer and van Dam, 2005; Van Dam, 2009; Van Dam and Heil, 2011; Soler et al., 2012).

Given that plants may have to respond to variable stressors in both the AG and BG compartments, it is somewhat surprising that the importance and significance of interactions between these compartments has only emerged in the past 20 years or so. For example, studies by Gange and Brown (1989) and Masters and Brown (1992) showed that root herbivory by a root chewing insect was positively correlated with the pupal weight of a leaf mining insect. Masters (1995) found that leaf mining AG significantly decreased the performance of chafer larvae feeding BG, but at the same time root herbivory increased the pupal weight of the leaf miner. This positive influence of root feeding can also influence higher trophic levels. For instance, the abundance of a seed predator and two of its parasitoids were highest on thistle plants subjected to root herbivory (Masters et al., 2001).

It is now known that organisms in both compartments can indirectly influence each other through changes in the biomass, nutritional quality (primary metabolites) and chemical defense (secondary metabolites) of plants (Bezemer and van Dam, 2005; Van Ruijven et al., 2005). Recently, Kostenko et al. (2012) reported that in ragwort (*Jacobaea vulgaris*) herbivory by AG- and BG-feeding herbivores affects the soil fungal community, which in turn affects plant defense, biomass, and multitrophic interactions in ragwort plants in successive generations in different years. In many plant taxa secondary plant compounds are produced in the roots and then transported to AG plant structures (Karban and Baldwin, 1997). Besides defense compounds, levels of nutritional metabolites, such as amino acids and carbohydrates, are often also affected by damage (Bezemer and van Dam, 2005). The capacity of roots to absorb nutrients and the chemical composition of the soil are strongly affected by soil organisms. This affects the growth rate of plants, which is important in structuring plant communities and associated organisms (Van Dam and Heil, 2011).

Differences in physical characteristics of the AG and BG compartments may have profound effects on the spatial and temporal processes and scales that shape interactions between plants and associated organisms across several trophic levels. It is generally far easier for plant antagonists and mutualists to disperse in the AG than in the BG domain, since movement is clearly much more limited BG as a result of the simple physical difference between air and soil. For example, in the AG compartment, herbivores generally have easy access to plant parts, such as shoots and flowers, possibly resulting in intense short-term selection for defense-related (or in the case of pollinators, attraction-based) traits (Zangerl and Berenbaum, 1993; Majetic et al., 2009; Parachnowitsch et al., 2012). As a result of these differences in the scale of AG–BG interactions, plants may have evolved variable responses to organisms in each compartment based on time differentials in the temporal sequences (and/or accumulative effects) of these antagonists. This temporal differential may lead many plants to evolve strong AG defenses whereas they have evolved to “tolerate” their BG antagonists until some critical threshold is reached whereby a plant population is forced to relocate to a new habitat (the “above-ground selection, below-ground dispersal hypothesis”; Van der Putten et al., 2001; Bezemer et al., 2005). The release and perception of chemical cues, such as herbivore-induced plant volatiles (HIPVs), may also reflect differences between the AG and BG compartments. The rate and extent of transport of these cues are likely to be reduced BG. Furthermore concentrations of unspecialized and more specialized compounds may also differ between AG and BG plant tissues, and the dependency of chemical communication on water-soluble compounds is likely to be greater BG (Van der Putten et al., 2001).

Interactions between consumers in the AG and BG compartments have been very well studied in recent years (Wurst, 2010; Anderson et al., 2011; Soler et al., 2012, 2013). Many of these studies have focused on elucidating mechanisms involving AG and BG organisms sharing the same plant (Erb et al., 2008). These interactions may vary in terms of complexity and may involve organisms from several trophic levels and functional groups or feeding guilds. Moreover, different genotypes of one plant species can differ in their response to BG or AG organisms (Wurst et al., 2008; Harvey et al., 2011), and BG and AG organisms themselves can respond differentially to plant genotypes (Crutsinger et al., 2006; Johnson, 2008; Kabouw et al., 2011; Utsumi et al., 2011). The variable responses both in the plant and the herbivore make it difficult to predict the outcome of AG–BG plant-mediated interactions. For example, although plant genotype correlated positively with AG and BG invertebrate colonization, correlations between the AG and BG invertebrate groups themselves were negative, suggesting that the two groups selected plant genotype differentially (Vandegehuchte et al., 2011).

The importance of higher trophic levels on herbivore-plant interactions was recognized first by Price et al. (1980). Since AG–BG interactions may occur between organisms across several different feeding guilds and species, it is not surprising to find that the outcomes of these interactions may vary substantially from one association to another. Most AG–BG interaction experiments to date have focused on the effect of BG organisms on AG organisms, but there are also some studies that have looked at the effect of AG on BG (or both; for a more in-depth discussion, see reviews by Soler et al., 2012, 2013). Plant-mediated AG–BG interactions may be decidedly non-linear, whereby small scale interactions between a plant and one type of organism can affect entire AG and BG food webs and communities associated with that plant (Bardgett and Wardle, 2003; Wardle et al., 2004, 2005; De Deyn et al., 2007; Gerber et al., 2007; Heil, 2011).

Several hypotheses have been proposed on the underlying mechanisms determining plant-mediated AG–BG interactions. The stress response hypothesis states that the removal of root biomass by root feeding organisms causes a similar response as drought stress (Masters et al., 1993). This results in an accumulation of soluble nitrogen and carbon in aboveground plant parts, thus increasing the nutritional quality of the plant for AG herbivores (Masters and Brown, 1997). By contrast, the defense induction hypothesis posits that herbivores in the opposite compartments negatively influence each other through induction of toxic secondary plant compounds (Bezemer et al., 2003; Bezemer and van Dam, 2005). Because these compounds are often stored in the cells, phloem feeders will be less exposed to inducible toxic compounds, perhaps explaining why root feeders often negatively influence the performance of leaf chewers but not that of aphids. On the other hand, AG herbivores may negatively affect the growth and development of BG herbivores by reducing the availability of carbohydrates in the roots (Van der Putten et al., 2001). Using cotton plants, Bezemer et al. (2003) found no effect of previous feeding by a leaf chewing caterpillar *Spodoptera exigua* on the performance of root feeding *Agriotes lineatus *larvae. On the other hand, they found that root feeding by wireworms negatively affected the performance of *S. exigua*. Wurst et al. (2006) looked at the effect of two soil organisms on primary and secondary metabolites in cabbage and found that foliar concentrations of glucosinolates, secondary metabolites characteristic for brassicaceous plants, were affected by these organisms. Earthworms decreased the concentration of glucoiberin in the plant shoots and interactions between earthworms and root-knot nematodes in turn affected concentrations of glucoraphanin. This may have an influence on AG herbivores, since glucoiberin can act as a feeding and oviposition stimulant, providing support for the defense induction hypothesis (Wurst et al., 2006). Another study also found a negative impact of root feeding on the oviposition and feeding behavior of an aboveground herbivore (Anderson et al., 2011). More studies showed that root herbivory, through reduced plant quality, negatively affected the performance of AG herbivores, parasitoids and even hyperparasitoids (Van Dam et al., 2004a; Soler et al., 2005). AG herbivory by caterpillars of the large cabbage white butterfly, *Pieris brassicae*, negatively affected performance of a root feeding herbivore, the cabbage root fly, *Delia radicum*, and its endoparasitoid, *Trybliographa rapae* (Soler et al., 2007a). Infestation of pepper plants with whiteflies elicited a BG defense response, resulting in reduced infection when exposed to AG and BG bacterial pathogens, whilst positively affecting the association of plant roots with beneficial micro-organisms (Yang et al., 2011). 

Not only are plant-mediated AG–BG interactions modified by the feeding activity of arthropods, but also by the composition of the soil micro-fauna. A meta-analysis of studies investigating the effect of mycorrhizal fungi on the performance of insect herbivores showed that the mycorrhizal status of host plants is often ignored in studies, despite the fact that mycorrhizal fungi can induce morphological, physiological, and biochemical changes and thus may influence plant quality for herbivores (Koricheva et al., 2009). In general, mycorrhizal fungi provide plants with nutrients and water and in return receive carbohydrates from the plant. The meta-analysis also revealed that phloem feeders benefited from mycorrhiza, whereas mesophyll feeders did not. The effect of dietary specialization in combination with feeding mode was only significant for the chewing and not for sucking sucking herbivores: specialist chewing herbivores performed better on plants colonized by mycorrhiza, whereas generalist chewing herbivores performed more poorly. In addition, mycorrhiza affected chewing herbivores negatively when these herbivores were feeding on the roots (Koricheva et al., 2009).

Bezemer et al. (2005) showed that the soil community composition can influence AG multitrophic interactions by affecting plant nutritional quality. Inoculation with nematodes negatively affected aphid offspring production, and aphid population size was lowest in microcosms with both nematodes and microorganism. The reverse was found for the aphid parasitoids which performed best in microcosms with both nematodes and microorganisms (Bezemer et al., 2005). These examples clearly illustrate that there are many different outcomes that may be generated by AG–BG interactions.

### DEFINING DIFFERENT TYPES OF GENETIC VARIATION

Genetic variation can be studied at various levels of organization, from the expression of genes at the molecular level to variation in traits at the organismal level. Here, we have focused on genetic variation at the level of the individual plant. According to Whitham et al. (2003), in order to better understand interactions between species and communities, genetic variation should be divided in three classes: (1) genetic variation within single populations of the same species, (2) genetic variation between different populations of the same species, and (3) genetic variation among different species. Genetic variation is known to be expressed in many different plant traits, including morphology, phenology, primary and secondary chemistry. The expression of specific secondary metabolites is often taxonomically constrained (Schoonhoven et al., 2005). For example, different plant families are often characterized by their own classes of secondary metabolites, e.g., alkaloids in the Solaneceae, benzoxazinoids in the Poaceae, and glucosinolates in the Brassicaceae.

Intra-specific genetic variation in plant AG defense traits and its effects on the behavior and/or development of herbivores and their natural enemies in both lab and field studies has been well studied. In particular, much is known about this field of research in cultivated and wild plant species in the Brassicales, which includes cabbages, mustards, and related crops and their wild relatives. This includes *Arabidopsis thaliana* (Bidart-Bouzat and Kliebenstein, 2008; Wentzell and Kliebenstein, 2008), *Brassica nigra* (Lankau and Strauss, 2007, 2008), *B. rapa* (Pilson, 1996, 2000), *Raphanus raphanistrum* (Agrawal et al., 2002), and both wild (Harvey et al., 2007, 2011; Gols et al., 2008a,b; Newton et al., 2009a,b) and cultivated (Poelman et al., 2008; Kos et al., 2011) *B. oleracea*. These studies and others with different plant taxa have generated a wealth of mechanistic data showing the reciprocal effects of genetic variation in AG plant defense traits on consumers up the food chain, as well as both biotic and abiotic factors that may be driving this variation (Crutsinger et al., 2006; Johnson, 2008; Newton et al., 2009a; Utsumi et al., 2011).

Genetic variation is usually based on trade-offs involving the costs and benefits of retaining certain traits when metabolic resources are limiting (Stearns, 1992). For example, trade-offs may occur in resource allocation between defense traits and growth (e.g., competitive ability). This has been reported in a number of invasive plants when released from their co-evolved native enemies (e.g., pathogens and herbivores) in their new ranges. In this situation, plants quickly reallocate metabolic resources from defense to growth, meaning that they are able to out-compete native vegetation (Wolfe et al., 2004; Zangerl and Berenbaum, 2005). This rapid switch from defense to growth supports the predictions of the “enemy-release” and “evolution of increased competitive ability” hypotheses (Maron and Vila, 2001; Keane and Crawley, 2002; Colautti et al., 2004; Joshi and Vrieling, 2005). Within the group of plant defense traits, there are also numerous trade-offs. The different defense traits of plants may conflict because of their energy demand (Van der Putten et al., 2001).

Storing valuable resources in the roots can make a plant less attractive for AG herbivores, but it will make the roots more attractive to BG herbivores. Re-allocating resources from roots to shoots and leaves may affect resistance to AG herbivory, but also means limited capacity of the roots to establish/maintain mutualisms with BG microorganisms (Heil, 2011). Among populations, different plant traits can be selected for, depending on the local conditions. The resulting local adaptation means that individual plants have a higher fitness at their home site compared to other sites inhabited by the same species (Kalske et al., 2012). Trade-offs in local adaptation can be caused by limited resources, allocation costs, or ecological or genetic constraints (Kalske et al., 2012). Thus far, trade-offs in various defense related traits in plants in response to combined AG and BG biotic interactions has received little attention, and therefore is a fertile area for future research (but see Vandegehuchte et al., 2011). It is important to keep in mind that various plant traits are not necessarily costly to maintain or, conversely, only have weak (or no) effects on plant fitness, in which case it is unlikely that adaptation will occur.

### GENETIC VARIATION IN DEFENSE AND OTHER TRAITS IN PLANTS

Evolution can only take place when natural selection acts on genetic variation in heritable traits that affect fitness (Whitham et al., 2003; Hughes et al., 2008). Without heritable phenotypic variation, there is no adaptive evolution possible. It is therefore important to determine what factors generate and maintain genetic variation within and between different populations (Siepielski and Benkman, 2009). Important sources for genetic variation in plants are introgression, mutation, and recombination at the gene level (Siepielski and Benkman, 2009), and also gene flow and genetic drift at the population level. The fact that there is heritable trait variation does not automatically mean that different levels of genetic diversity have predictable ecological consequences, because other factors (e.g., the environment) also play an important role (Hughes et al., 2008).

Genetic variation in plant defense traits is driven by a number of biotic and abiotic factors that may well be synergized (see discussion below with wild cabbage to get a better perspective). Much attention has been paid to trophic interactions between plants and their antagonists such as pathogens and herbivores, often in a co-evolutionary framework. Indeed, co-evolutionary theory underpins our understanding of intimate consumer-resource interactions in nature (Pimentel, 1961; Ehrlich and Raven, 1964; Rosenzweig, 1973; Abrams, 1986; Marrow and Cannings, 1993; Bonte et al., 2010; de la Peña et al., 2011). Many of the classical studies on co-evolutionary arms races and adaptive radiation have explored interactions between insect herbivores and their food plants (Ehrlich and Raven, 1964; Benson et al., 1975; Berenbaum and Zangerl, 1992; Hamrick and Godt, 1996; Pilson, 1996, 2000; Janz and Nylin, 1998; Lankau, 2007; Lankau and Strauss, 2007; Becerra et al., 2009; Cogni and Futuyma, 2009; Carmona et al., 2011; Bode and Kessler, 2012; Holeski et al., 2012; Bernhardsson et al., 2013). More recently it has been argued that selection for certain traits occurring in a pair-wise fashion are often generated at local or small landscape scales, and the term evolutionary “hotspots” has been invoked to describe this phenomenon (Thompson, 2005a). In this situation localized populations of closely interacting species interact intensively in small, often isolated patches and thus evolve unique traits that reflect adaptations to one another: one (the consumer) to exploit and one (the resource) to resist. Given that selection intensity can vary depending upon local conditions, evolutionary hotspots may be distributed over space and time as “geographic mosaics” (Thompson, 2005a). Thus far, however, discussion of selection pressures generated in hotspots has focused on the AG domain, with little effort to determine if and to what extent selection can occur from combined AG–BG interactions. To study this it is necessary to measure genetic variation in the expression of AG–BG plants traits and to determine if they are correlated (see e.g., Kaplan et al., 2008). Moreover, field studies are needed to identify and measure qualitative and quantitative differences in AG and BG communities associated with a plant at different spatial and temporal scales. In addition, selection on certain plants may be characterized by diffuse selection (Strauss and Irwin, 2004; Vandegehuchte et al., 2011). Alternatively, herbivores may respond to variation in defense traits without exerting any selection pressures themselves.

As described above a plethora of studies have examined the biotic factors driving selection for AG defense traits in plants, and in particular allelochemistry (Coley et al., 1985; Schoonhoven et al., 2005). For example, Zangerl and Berenbaum explored whether herbivores can select for rapid increases in secondary metabolites (xanthotoxins) in plants, using wild parsnip and its main herbivore, the parsnip webworm, *Depressaria pastinacella* as a model system. This plant species harbors few herbivores in nature, aside from *D. pastinacella*, whose larvae attack seeds and thus may greatly affect plant fitness (Zangerl and Berenbaum, 1993). Wild parsnip has been introduced into various parts of the world where it has become an invasive pest in some areas (Berenbaum et al., 1986). In some regions where it has been introduced, webworms have also been released as a means of biological control, although in many habitats where the plant is established these herbivores are still absent. The main secondary metabolites in *P. sativa* are furanocoumarins, toxic compounds found primarily in species of the Apiaceae and Rutacea. Zangerl et al. (2008) showed that in areas where webworms are absent, parsnips rapidly responded by reducing investment in chemical defenses, suggesting that they are costly to maintain (see also Berenbaum and Zangerl, 2006). However, when webworms were introduced into regions where parsnips had been established for some years, the plants rapidly responded by reallocating metabolic resources to the production of furanocoumarins, showing that rapid evolutionary responses to chemical defenses are possible (Berenbaum and Zangerl, 1998).

The role of higher trophic levels, such as predators and parasitoids, in driving selection of plant-related traits has received less attention, although it has been amply demonstrated that natural enemies can significantly reduce herbivore abundance in agricultural landscapes (Luck et al., 1988; DeBach and Rosen, 1991). Cropping systems are often characterized by monocultures of plants whose direct chemical defenses have been greatly reduced as a result of artificial selection via domestication (Gols and Harvey, 2009). Natural systems are generally much more complex than agro-ecosystems chemically and structurally. How important trophic cascades involving insects are, has been the subject of debate (Hairston et al., 1960; Huntly, 1991; Schmitz et al., 2000). Evidence is coming to light that investment in costly plant secondary metabolites can be significantly influenced by the presence or absence of parasitoids, and that these effects generate phenotypic mosaics at the landscape-scale. Once again, the best studied system in which this area has been explored is the *P. sativa*–*D. pastinacella* association. Work by Berenbaum, Ode and colleagues has found that one parasitoid species, the encyrtid wasp *Copidosoma sosares*, devastates *D. pastinacella *populations where both species along with the food plant are native in western and central Europe (Ode et al., 2004; Berenbaum and Zangerl, 2006; Ode, 2006; Lampert et al., 2008). Where all three species are common in the native range, *P. sativa* plants are apparently less toxic than in areas of the invasive range where only the plant and herbivore have been established (Berenbaum and Zangerl, 2006). However, when plants and herbivores in the invasive range have been reunited with *C. sosares*, the plants quickly lower investment into the production of furanocoumarins, presumably because the parasitoids are again greatly reducing levels of herbivory (Berenbaum and Zangerl, 2006). Future studies comparing defense traits in populations of native and invasive plants in a multitrophic framework incorporating natural enemies offer much promise in better understanding rapid shifts in traits, such as from defense to growth. More importantly, future studies need to explore this combining AG and BG compartments, given what we already know about the importance of this linkage.

Where herbivores might select for high chemical defense levels, competition between plants might select for other plant traits although some of these may also involve phytotoxins (e.g., in the case of allelopathy). This is a complex matter, since plants compete not only with other plant species but also with conspecifics. For example, sinigrin produced by *B. nigra *is allelopathic and retards the germination and growth of wild oat and wild barley (Tawaha and Turk, 2003; Turk and Tawaha, 2003). Lankau and Strauss (2008) stated that, due to the costs of trait maintenance, a trait that improves interspecific competition will at the same time reduce intraspecific competition. Sinigrin is costly to produce and functions not only in competition with other plants but also as a defense compound against herbivores and pathogens.

In *B. nigra* there is a negative genetic trade-off between inter- and intraspecific competitive ability (Lankau and Strauss, 2008). When competing with different plant species, genotypes that produced high concentrations of sinigrin were strong competitors. However, in competition with conspecifics, these genotypes did poorly because there were no benefits to be gained from producing high concentrations of sinigrin (Lankau and Strauss, 2008; Lankau et al., 2011). While interspecific competition is primarily influenced by the allelopathic and antimycorrhizal effects of sinigrin, intraspecific competition is based on resource capture (Lankau and Strauss, 2007). Lankau and Strauss (2008) also found that the assembly of plant and herbivore species present in a community influence selection pressures acting on the production of sinigrin. Importantly, the identity of the competing plants affected selection for sinigrin production in the presence of herbivores more than the number of neighboring plants. Thus, the associated plant and herbivore community acts as an important and variable selection pressure on sinigrin in black mustard.

Johnson et al. (2008) studied the effect of plant genotype on intra-specific competition between evening primrose (*Oenothera biennis*) plants. This plant species exhibits heritable variation in above- and belowground growth and different genotypes responded differently to competition. Although evening primrose affected other plants through competition in the greenhouse, it was found that soil fertility had a much stronger effect and that in the field, there was no genotypic effect on neighboring plants. They concluded that in this case environmental variation was a stronger determinant of competition than plant genotype (Johnson et al., 2008).

Although this area has been little studied, we argue that interactions between plants and AG and BG organisms may influence the evolution of traits such as defense against herbivores, attraction of pollinators, as well as competition between plants for access to water, nutrients and light. The way these interactions are played out in natural communities can affect plant fitness. For example, Poveda et al. (2003) looked at the separate and combined effects of root and leaf herbivory on plant fitness in charlock mustard, *Sinapis arvensis*. Root herbivory marginally increased the flowering period and number of fruits produced when compared with combined root and leaf herbivory. It was also correlated with a higher *per capita* number of flower visits by pollinators (Poveda et al., 2003). These finding are in contrast with other studies where a negative effect of root herbivory on plant growth (Gange and Brown, 1989) or reproduction (Masters et al., 2001) was found, although in the Poveda et al. (2003) study this may have been caused by the low number of root herbivores per plant.

Barber et al. (2011) examined the effect of AG and BG herbivory on the performance of cucumber plants and found that, although root herbivory positively affected flower visitation by honey bees, root herbivory had a stronger negative effect on plant reproduction than leaf herbivory. Moreover, plant growth was reduced by both leaf and root feeding, whereas flower production was negatively affected by root herbivory resulting in less female flowers. Maron (1998) studied the effects of AG and BG insect herbivores on bush lupine and found that they had the potential to impose strong selection on the plant in several ways: via the suppression of AG herbivores that increased seed production and via the suppression of BG herbivores that also increased seed production but which additionally decreased plant mortality. The results of these experiments shed some light on the complexity of AG–BG interactions and plant fitness and suggest that in terms of selection regimes the effects may not only be association-specific but also vary in different populations at the landscape scale.

Not only do herbivores exert selection pressures on plants, but it also works the other way around. Because plants and specialist herbivores are often involved in a co-evolutionary arms race, anti-herbivore defenses of plants may select for herbivore genotypes that are best able to deal with those defenses and vice versa (Kant et al., 2008). For example, spider mites dealing with jasmonic acid in tomato plants developed three different genotypes that differentiated in their induction of and resistance to jasmonic acid-induced defenses (Kant et al., 2008). Some populations of a perennial herb had associated herbivores that were locally adapted to their genotype, but in other populations the plants were adapted to the herbivores (Kalske et al., 2012). It remains to be determined at larger spatial (=geographical) scales if differences in combined AG–BG selection pressures can drive genetic variation in plant responses at the species and population level, as reflected in the measurement of different traits such as growth and defense. Moreover, how AG and BG plant responses in combination can drive reciprocal selection in herbivores and perhaps even their natural enemies is largely unknown (but see Bonte et al., 2010; Vandegehuchte et al., 2011). Although this infers the expression of some immensely complex processes that span several to many links, there is no reason that such effects do not occur in habitats where there are strong frequency-dependent AG and BG interactions.

### DEFENSE CHEMISTRY IN WILD CABBAGE, *Brassica oleracea*, AND RELATED SPECIES

Plant species in the Brassicaceae are well studied with respect to (genetic) variation in secondary plant chemistry (Halkier and Gershenzon, 2006; Agerbirk and Olsen, 2012) and their interactions with AG and BG insect herbivores, but also with species in the third and even the fourth trophic level (Harvey et al., 2003; Soler et al., 2005, 2007b; Gols and Harvey, 2009; Hopkins et al., 2009). Secondary metabolites characteristic for plants in this family are the glucosinolates (hereafter GS). They are sulphur- and nitrogen-containing plant secondary metabolites that can be divided in three different classes based on their amino acid origin: aliphatic, indolyl and aromatic GS (Halkier and Gershenzon, 2006). When plant tissues are disrupted by for example insect feeding, myrosinase enzymes come into contact with the intact GS, and hydrolyze them into various hydrolysis products. Especially these GS breakdown products play a role in defenses against various attackers such as generalist insect herbivores and pathogens (Mithen, 2001). However, as specialist insect herbivores have evolved efficient mechanisms to excrete, detoxify or sequester GS (Bridges et al., 2002; Ratzka et al., 2002; Wittstock et al., 2004; Müller, 2009a), they may use GS and their breakdown products as stimuli to recognize host plants for oviposition and feeding (Renwick, 2002; Renwick et al., 2006; Bidart-Bouzat and Kliebenstein, 2008). Plant quality for specialist herbivores is determined by more general plant characteristics such as levels of primary metabolites and mechanical traits (Travers-Martin and Müller, 2008).

**FIGURE 1:**
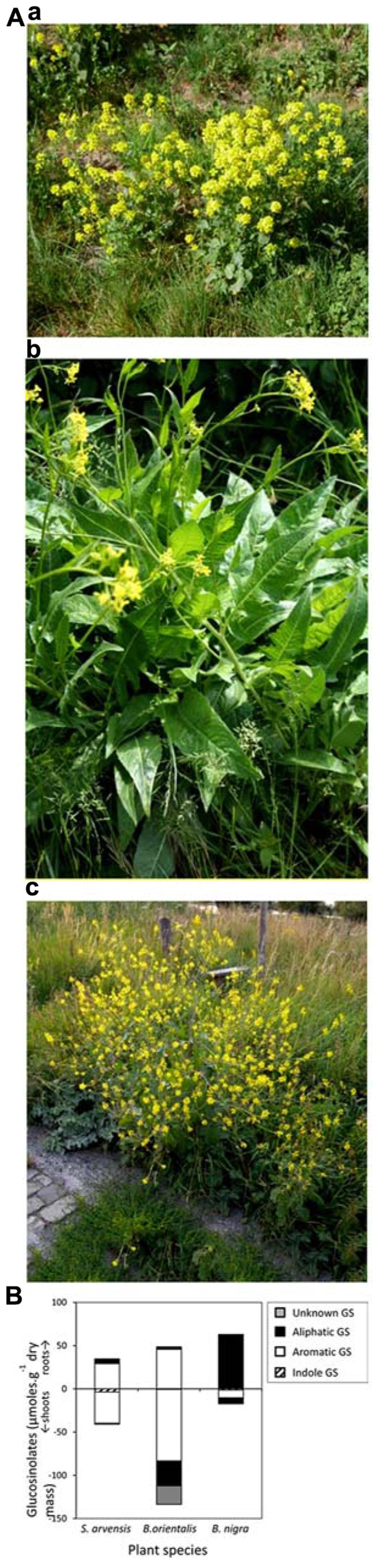
**(A) ***Sinapis arvensis* (a), *Bunias orientalis* (b), and *Brassica nigra* (c). **(B)** Mean (*n* = 10) shoot and root (negative values) glucosinolate levels in undamaged greenhouse-grown *Sinapis arvensis, Bunias orientalis, Brassica nigra* originating from natural growing populations in the Netherlands (Glucosinolates were classified according to their amino acid origin into indole, aromatic and aliphatic GS. The dominant GS in *S. arvensis* and *B. orientalis* was the aromatic GS sinalbin, whereas the dominant GS in *B. nigra *was the aliphatic GS sinigrin. The root tissues of *B. orientalis* also contained relatively high levels of an unknown GS. (see Van Dam et al., 2004b for analysis methods).

**FIGURE 2:**
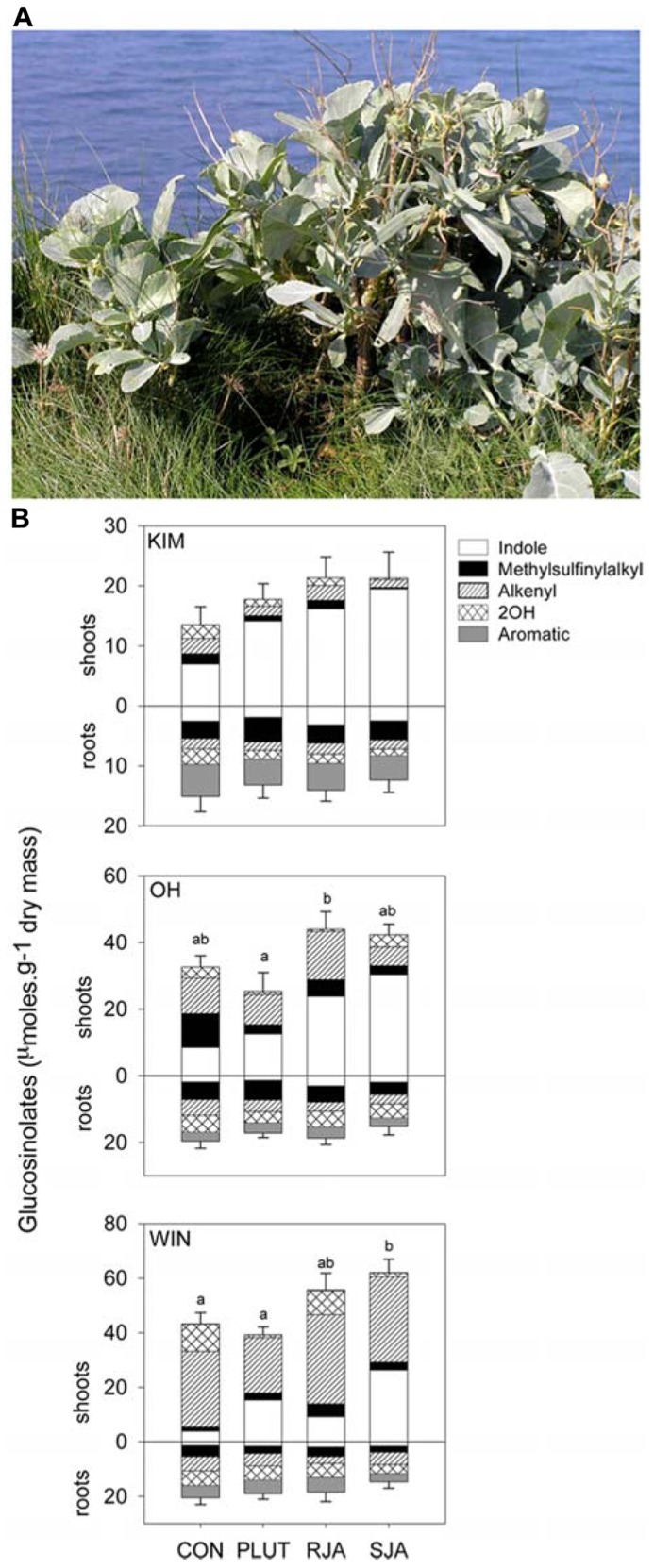
**(A)** *Brassica oleracea*. **(B)** Shoot and root glucosinolate levels (mean + SE of mean total, *n* = 4 or 5) of *Brassica oleracea* plants originating in Dorset, England from three wild populations located at sites called Kimmeridge (KIM), Old Harry (OH), and Winspit (WIN), respectively. Glucosinolates were classified according to their amino acid origin into indole, aromatic and aliphatic GS. The latter group was further divided into methylsulfinyl, alkenyl, and hydroxyl (=OH) GS. The plants were either untreated controls (CON), induced with 9 second instar *Plutella xylostella* (PLUT) larvae divided over three leaves, induced with 500 μg jasmonic acid either applied to the roots (RJA) or to the shoots (SJA). Jasmonic acid was used to simulate herbivory by chewing herbivores (Van Dam et al., 2004b). Roots and shoot tissues were harvested for GS analysis 7 days after the induction treatments. Different letter over the bars indicate significant differences (*p* < 0.05) in total glucosinolate level between the bars within each panel (Tukey HSD multiple comparisons among means). Please note the difference in scaling of the Y-axes. Both population and induction treatment had a significant effect on total GS levels in wild *B. oleracea* (MANOVA, treatment *F*_6,82_ = 5.77, *p* < 0.001; population *F*_4,82_ = 18.7, *p* < 0.001). All classes of GS, as well as total GS concentrations, differed with population origin in both the roots and the shoots (*p *< 0.05 for all analyses). In the shoots, indole GS (*F*_3,__42_ = 23.9, *p* < 0.001) increased in response to the three induction treatments. Aromatic GS were also affected by induction treatment (*F*_3,42_ = 3.34, *p* = 0.03). Only WIN shoots contained small amounts of aromatic GS and these decreased with shoot induction, *P. xylostella* feeding and JA treatment, but increased with root JA application. In the roots, only indole GS responded significantly to induction treatment (*F*_3,42_ = 7.57, *p* < 0.001); JA applied to the roots increased indole GS levels in these tissues.

Various species of *Brassicas* differ in their GS profiles, both in AG and BG tissues (**Figures 1** and **2**). For example, the relative GS concentrations in AG and BG tissues differ dramatically, with root concentrations being much higher than shoot concentrations in *Bunias orientalis*, these being lower in* B. nigra *and similar in *S. arvensis *(**Figure 1B**). Usually, levels of GS are lower in BG than in AG tissues (Van Dam, 2009). Across species, variation in defense chemistry has been demonstrated to affect the performance of associated insects (Francis et al., 2001; Müller et al., 2002; Renwick, 2002; Harvey et al., 2003, 2010; Gols et al., 2008c). These dramatic differences in plant secondary chemistry at the species level may have implications for the interactions with other organisms in nature.

Although GS have been shown to play an important role in protecting plants against generalist insect herbivores (Blau et al., 1978; Gols et al., 2008a), other studies have shown that this is not always the case. For example sinalbin, the dominant GS in both AB and BG tissues in *B. orientalis* (**Figure 1B**) appears not to be effective against feeding by the generalist herbivore *Mamestra brassicae* (Harvey and Gols, 2011). Moreover, the high GS concentrations in BG tissues in this invasive species may play a role in its competitive abilities with other plant species or soil organisms that negatively affect growth and development of this plant and may explain its invasion success, but this needs to be tested empirically (Müller, 2009b). Remarkably, *B. orientalis*, which is readily accepted for oviposition*,* is a poor food plant for all studied specialist herbivores (Harvey et al., 2010). This result suggests that other chemicals in *B. orientalis* render these plants unsuitable for development of specialist herbivores.

*Brassica oleracea* is native to the coastlines, especially calcareous cliffs, of Western Europe and is considered the progenitor of cultivated cabbage (Mitchell and Richards, 1979). In the UK, the largest populations are on the south-west coast in the counties Cornwall, Devon, and Dorset (Wichmann et al., 2008). The distribution of the wild cabbage populations along the Dorset coast has been very constant over the past 70 years (Wichmann et al., 2008). These populations have also been the subject in a number of studies investigating the variation in GS metabolites, as well as the factors that maintain this variation considering that these populations grown often less than 15 km apart. The differences in GS profiles are most likely caused by divergent abiotic and biotic selection at the different sites (Mithen et al., 1995; Moyes et al., 2000; Moyes and Raybould, 2001; Newton et al., 2009a, 2010), although random processes such as founder effects and genetic drift may have played a role here as well.

Variation in the expression of GS is not only expressed across, but also within species. Population of wild cabbage differ considerably in their GS profiles (**Figure 2**) with concomitant consequences for the performance of insects in the second and third trophic level (Gols et al., 2008a; Harvey et al., 2011). Moreover, GS concentrations change in response to herbivory or simulated herbivory (see also Van Dam et al., 2004b) and population related differences in induction, although not found here (**Figure 2B**), have been reported for the wild cabbage populations when induced by a different herbivore *Pieris brassicae *(Harvey et al., 2011). The variation in GS concentration appears to be more pronounced in AG than in BG tissues (**Figure 2B**). For example, indole GS dominate the profile in leaf tissues of the Kimmeridge (KIM) population, whereas leaves sampled from Winspit (WIN) plants contain high alkenyl GS concentrations. In the roots of the three populations all GS classes are represented and levels are relative little affected by induction compared to induction of foliar tissues.

Mithen et al. (1995) suggested that herbivores could act as an important selective force driving GS variation in the wild cabbage populations. However, Moyes et al. (2000) argued that for herbivores to act as a selection force, the herbivores need to select individual plants based on their GS profiles. They showed that there was a potential for host plant selection based on differences in the GS profiles of neighboring plants in the population on a small scale, but found no correlation between herbivore preference and GS profile except for one specialist herbivore species. They made the point that although laboratory experiments showed that GS influence the performance of herbivores, there was little evidence that this was also the case in nature (Moyes et al., 2000). In contrast, Newton et al. (2009a) reported significant differences in the response of herbivores to aliphatic GS, both within and between plant populations in the field. Based on their findings, they concluded that variation in GS can structure the associated herbivore community (i.e., herbivore mediated differential selection, Newton et al., 2009a). However, to demonstrate herbivore-mediated differential selection conclusively, further evidence is required showing that variable attacks by herbivores in the field have consequences for plant fitness (Newton et al., 2009a).

The populations along the Atlantic coast of England are known to be exposed to different abiotic conditions, despite their relative close proximity to each other. While some populations are located on high cliffs and thus are fully exposed to the prevailing wind, others are located in sheltered valleys. This may affect the colonization of plants by herbivores and their natural enemies, with populations on the cliffs experiencing low and population in the valleys experiencing high insect pressures. Soil characteristics such as clay and water content, soil texture, and nutrient levels have been reported to differ among the wild cabbage sites (Mithen et al., 1995; Wichmann et al., 2008). Little is known about the biotic selection pressures BG that may explain the relative lower variation in root GS chemistry of the wild cabbage populations. We currently investigate variation in associated soil communities at several of the wild cabbage sites in Dorset in order to reveal the degree of biotic BG variation.

The “Geographic Mosaic of Co-Evolution Theory” predicts that the intensity of selection pressure exerted by herbivores on plants may vary geographically (Thompson, 2005b). Local differences in selection pressure may thus result in population-related variation in the expression of certain traits. The ultimate question with respect to the wild cabbage populations is what processes maintain this high level of variation in secondary chemistry and potentially other traits and whether this variation is the consequence of strong selection pressures exerted locally. In addition, selection pressures may differ with respect to the AG and BG compartment. In other words, spatial heterogeneity in defense traits may be expressed differentially in AG and BG tissues as a result of differences in selection pressures in the two compartments. Moreover, the third trophic level as a selection force BG should be included as well (Price et al., 1980). In agricultural fields, cabbage root flies (*Delia radicum*) cause considerable damage to cabbage crops and they are also known to be attacked by various parasitoids species. Foraging behavior of parasitoids of *Delia radicum* has been reported to be affected by caterpillar feeding AG (Pierre et al., 2011). These results suggest the importance of a holistic approach of AG–BG multitrophic interactions.

## CONCLUSION AND FUTURE DIRECTIONS

The study of AG–BG multitrophic interactions is now a major area of research in ecology. Over the past two decades a significant amount of empirical data has demonstrated the importance of AG–BG interactions in terms of mechanisms relating to the behavior and development of insects and other invertebrates, as well as effects on community structure and food webs. As the field continues to blossom, it is hoped that links between AG and BG compartments can be used to explain important applications in ecology, such as the production and delivery of important provisioning ecological services, e.g., the maintenance of soil fertility, nutrient cycling, pollination, and even regional climate control. There is little doubt that a more intensive multi-disciplinary approach to the study of AG and BG ecology will yield many insights into the functioning of ecological systems and their role in sustaining human civilization.

At present, however, there are still some significant gaps in our knowledge of important mechanisms and processes, such as in the spatio-temporal variation in AG–BG interactions and in how they may drive selection for different plant-related traits such as defense and competitive ability. Furthermore, we are only beginning to scratch the surface in our understanding and appreciation of the role played by natural enemies in generating variation in various plant traits. Given the potential importance of trade-offs between tolerance (growth) and defense in plants, the influence of natural enemies such as parasitoids in driving selection may be vastly underappreciated. If we incorporate natural enemies of plant antagonists in the soil, and then link these with three or even four trophic level interactions AG, there is a potential wealth of outcomes that remains to be explored. Moreover, given what we now know about evolutionary hotspots where selection is played out intensively, it would be interesting to search for these hotspots in a plant species within and between habitats, and to try and match phenotypes with strong AG, BG, and combined (AG and BG) selection regimes. Furthermore, given that plants can also potentially drive genetic variation in their associated consumers over several trophic levels and via multiple linkages, it would be interesting to explore how this may be played out combining AG and BG interactions.

We suggest several areas for future investigations:

(1) Studies working with different genotypes of wild plants and determining how these affect the behavior and performance of AG and BG insect herbivores and their natural enemies associated with them both independently and in combination;
(2) Analyzing various plant traits in roots and shoots in the same plant species both within and between populations along a geographical transect where abiotic and biotic selection pressures may vary. Furthermore, working to determine how differences in these traits are correlated with selection pressures from antagonists in the roots and shoots;
(3) (3) Searching for geographical “hot-spots” in which selection for AG and BG responses are rigidly enforced and the interactions with the various consumers are identified;
(4) (4) Comparing AG and BG interactions in geographically widespread plants both in the native and invasive ranges, and determining how release from their co-evolved natural enemies AG, BG (or both) may have led to a relaxation in selection for defense-related traits. Studies with invasive plants have generally ignored links between AG and BG trophic interactions, which may be a major omission in understanding why a small percentage of exotics become invasive pests.

In summary, we argue that the field of AG–BG multitrophic interactions needs to explore a wider range of biotic and abiotic selection pressures in explaining genetic variation in plant-related traits (and also reciprocally in their consumers up the food chain). In doing so it will be possible to develop a more thorough appreciation of the questions underpinning the immense variation in traits expressed in plants at various spatial scales.

## Conflict of Interest Statement

The authors declare that the research was conducted in the absence of any commercial or financial relationships that could be construed as a potential conflict of interest.
